# Retrotransposon LINE‐1 Activation Drives Microglial Dysregulation in Late‐Onset Alzheimer's Disease

**DOI:** 10.1002/alz70855_102972

**Published:** 2025-12-23

**Authors:** Falak Sher

**Affiliations:** ^1^ Columbia University, New York, NY, USA; Taub Institute, New York, NY, USA

## Abstract

**Background:**

Microglia, the brain's resident immune cells, play a critical role in the pathogenesis of late‐onset Alzheimer's disease (LOAD). Recent evidence suggests that transposable elements such as long interspersed nuclear element‐1 (LINE‐1) may contribute to neuroinflammation and cellular dysfunction in LOAD. However, the specific role of LINE‐1 in microglial dysfunction and its contribution to disease pathogenesis remain poorly understood.

**Methods:**

We investigated LINE‐1 expression in neurons, astrocytes, oligodendrocytes, and microglia using postmortem prefrontal cortex tissue from individuals with LOAD and age‐matched cognitively normal controls. Immunoreactivity of the LINE‐1‐encoded open reading frame 1 protein (ORF1p) was quantified in microglia, and correlations with disease‐associated morphology were assessed. In vitro, human iPSC‐derived microglia (iMG) were used to model LINE‐1 activation via CRISPR‐mediated transcriptional activation. Microglial morphology, cytokine secretion, amyloid beta (Aβ) phagocytosis, and transcriptomic changes were analyzed.

**Results:**

We observed elevated ORF1p immunoreactivity in microglia from LOAD patients compared to controls, correlating with disease‐associated microglial morphology. In iMG, CRISPR‐mediated LINE‐1 activation induced morphological changes, altered cytokine secretion, and impaired Aβ phagocytosis. Transcriptomic analyses revealed that LINE‐1 activation affected genes involved in antigen presentation, lipid metabolism, and multiple Alzheimer's disease‐relevant pathways.

**Conclusions:**

Our findings suggest that LINE‐1 activation drives microglial dysregulation, contributing to neuroinflammation and cellular dysfunction in LOAD. These data identify LINE‐1 as a novel mechanism of microglial impairment and position it as a potential therapeutic target for mitigating neuroinflammatory processes in Alzheimer's disease.

